# On Some of the Signs of Incipient Pulmonary Phthysis

**Published:** 1854-02

**Authors:** 


On some of the Signs of Incipient Pulmonary Phthysis.—M. Bourdon says:—It is known with what difficulty the presence of pulmonary tubercles is diagnosticated when the disease is in its incipiency, and how insufficient the stethoscopic signs are. The latter may be wanting when the lungs contain a large quantity of tubercles; they may exist when the lungs contain none; and they may be masked by different sounds. The general symptoms are not a sure means of detecting the disease; a simple catarrh may be attended with very grave symptoms, while a true phthisis may affect the general system very slightly. It is at the commencement of the disease that the diagnosis is most important, because this is the only time that there is a chance of treating it with any success. M. Bourdon has sought to elucidate this obscure point. Attracted for a long time by the frequency of certain symptoms in phthisical patients, he has studied them with care, in order to establish their value as signs. These symptoms are arranged under many heads, according to the organs in which they show themselves:—1. Gastric symptoms. 2. Hepatic symptoms. 3. Thoracic pain. The gastric symptoms are not to be confounded with the morbid phenomena observed in almost all diseases. Those which coincide with the incipiency of phthisis are nauseas, vomitings, epigastric pains, dyspepsia. You must not wait to find these symptoms in every case; nevertheless, they are extremely frequent, since M. Bourdon found them in more than two-thirds of his subjects. Some phthisical patients suffer from one of these symptoms; others, on the contrary, have them all combined. Nausea and vomiting are most common; and, it is generally after the cough that they exhibit themselves. It must be observed, however, that their frequency or intensity are not at all proportioned to the cough; they occur when the stomach is empty as well as after a repast. Generally the vomitings consist of mucous matter, rarely of bile. Still, while they show themselves with a large number of the tubercles, they do so in a positive manner with very few. Another very common symptom is the epigastric pain. In the largest number of cases, it is accompanied with nausea and vomiting; nevertheless it occurs alone sometimes. With the largest number of patients it is only manifested upon pressure, or is so slight as not to be noticed by the patient until their attention is attracted to it; in a smaller number of patients it manifests itself spontaneously. Although it shows itself both before and after eating, it is yet most commonly observed after the ingestion of food. Long continued dyspepsia, without nausea or vomiting, is frequently observed. Frequently the gastric symptoms precede the thoracic; but often also they show themselves at the same time or subsequently. Do they imply a lesion of the stomach? is a question which has occupied the author. Sometimes he found the stomach healthy; most frequently it presented some lesion, which was oftenest a papillated state of the mucous membrane. The author thinks the pressure of the tumefied bronchial glands upon the pneumo-gastric might contribute to the disorders of the stomach.

2. The liver sometimes presents remarkable peculiarities. In half the patients examined by M. Bourdon there was an increase of volume, frequently attended with pain. The increase of volume seemed to be particularly observable in the right lobe; the consistence of the organ seeming to undergo no change. The changes of sensibility are less frequent than the changes of volume. Spontaneous pains are rare; they only manifest themselves under pressure, or under some sudden and straining movement. In some rare cases this change of sensibility is observed without change of volume. As to the change the liver undergoes, the autopsies prove that in more than half of the cases it is due to fatty degeneration more or less advanced. The bile is generally of greater density and more highly colored.

3. The largest number of phthisical patients suffer from pain between the shoulders and in the sides of the chest; but, besides these spontaneous pains, there are others to which M. Bourdon calls the attention of physicians, and which are only recognized through the medium of percussion. The points where these pains are discovered are numerous, but they are most often seated below the clavicles, and only on the side affected with tubercles. If both sides are affected the pain is on the side most affected. As to the cause of this pain, M. Bourdon, like M. Beau, is inclined to ascribe it to intercostal neuralgia.

The conclusions drawn by M. Bourdon from his researches are as follows:—When prolonged dyspepsia, nauseas and vomitings are observed, without any assignable cause; when the liver is abnormally developed, without there being hepatitis or disease of the heart; when these phenomena are exhibited independent of any other affection, or in the course of chlorosis, or after measles or typhoid fever; the physician should turn his attention to tuberculosis, examine the patient with great care, and even when there is doubt of the reality of the pulmonary affection, he ought to act as if convinced, or at least he should take his precautions.—*Archives Gen.—Virginia Med. and Sur. Journal.*

